# The impact of high-flow nasal cannula oxygen therapy on exercise capacity in fibrotic interstitial lung disease: a proof-of-concept randomized controlled crossover trial

**DOI:** 10.1186/s12890-020-1093-2

**Published:** 2020-02-24

**Authors:** Atsushi Suzuki, Masahiko Ando, Tomoki Kimura, Kensuke Kataoka, Toshiki Yokoyama, Eiichi Shiroshita, Yasuhiro Kondoh

**Affiliations:** 1 Department of Respiratory Medicine and Allergy, Tosei General Hospital, 160 Nishioiwake-cho, Seto, Aichi 489-8642 Japan; 2 Department of Respiratory Medicine, Nagoya University Graduate School of Medicine, Nagoya, Aichi Japan; 3 Center for Advanced Medicine and Clinical Research, Nagoya University Hospital, Nagoya, Aichi Japan; 4 Pacific Medico Co., Ltd., Chiyoda-ku, Tokyo, Japan

**Keywords:** Fibrotic interstitial lung disease, High-flow nasal cannula, Oxygen therapy, Exercise capacity, Pulmonary rehabilitation, Health status

## Abstract

**Background:**

Patients with fibrotic interstitial lung disease (FILD) often experience gas exchange abnormalities and ventilatory limitations, resulting in reduced exercise capacity. High-flow nasal cannula (HFNC) oxygen therapy is a novel treatment, whose physiological beneficial effects have been demonstrated in various clinical settings. We hypothesized that HFNC oxygen therapy might be superior to conventional oxygen therapy for improving exercise capacity in FILD patients.

**Methods:**

We performed a prospective randomized controlled crossover trial with a high-intensity constant work-rate endurance test (CWRET) using HFNC (50 L/min, FiO_2_ 0.5) and a venturi mask (VM) (15 L/min, FiO_2_ 0.5) for oxygen delivery in FILD patients. The primary outcome variable was endurance time. The secondary outcome variables were SpO_2_, heart rate, Borg scale (dyspnea and leg fatigue), and patient’s comfort.

**Results:**

Seven hundred and eleven patients were screened and 20 eligible patients were randomized. All patients completed the trial. The majority of patients were good responders to VM and HFNC compared with the baseline test (VM 75%; HFNC 65%). There was no significant difference in endurance time between HFNC and VM (HFNC 6.8 [95% CI 4.3–9.3] min vs VM 7.6 [95% CI 5.0–10.1] min, *p* = 0.669). No significant differences were found in other secondary endpoints. Subgroup analysis with HFNC good responders revealed that HFNC significantly extended the endurance time compared with VM (VM 6.4 [95%CI 4.5–8.3] min vs HFNC 7.8 [95%CI 5.8–9.7] min, *p* = 0.046), while no similar effect was observed in the VM good responders.

**Conclusions:**

HFNC did not exceed the efficacy of VM on exercise capacity in FILD, but it may be beneficial if the settings match. Further large studies are needed to confirm these findings.

**Trial registration:**

UMIN-CTR: UMIN000021901.

## Background

Fibrotic interstitial lung diseases (FILD) are progressive chronic lung diseases, including idiopathic pulmonary fibrosis (IPF) and other forms of ILD [1, 2]. Patients with FILD often experience exertion dyspnea and reduced exercise capacity, which leads to impairment of health status and poor prognosis [ 3–5]. Interventions to enhance exercise capacity and physical activity have an important role for the management of FILD patients.

Recently, there is a growing body of evidence that supplemental oxygen is effective in improving the exercise capacity of FILD patients. A previous double-blind, placebo-controlled, randomized crossover trial demonstrated that ambulatory oxygen did not improve exercise capacity and exertion dyspnea compared with placebo-air [6]. On the other hand, a recent prospective, open-label, crossover randomized controlled trial (AmbOx) showed that supplemental oxygen improves exercise capacity and exertional dyspnea compared with placebo-air in FILD patients [7]. Another randomized crossover trial also showed that supplemental oxygen provided through an oxygen conserving device improved endurance time and desaturation in FILD patients [8]. Based on these findings, short-burst supplemental oxygen during exercise is becoming common practice for FILD patients [9].

The high-flow nasal cannula (HFNC) is a novel device delivering heated and humidified oxygen via a nasal cannula at a maximum flow of 60 l/min.

This generates low levels of positive pressure in the upper airways and decreases physiological dead space by flushing out expired carbon dioxide.

Its beneficial effects and utility have been widely demonstrated in various clinical settings [10–13]. However, few studies have assessed the efficacy of HFNC on exercise capacity in patients with FILD.

We hypothesized that HFNC oxygen therapy might be superior to conventional oxygen therapy for improving exercise capacity in FILD patients. To assess this hypothesis, we performed a proof-of-concept prospective randomized controlled crossover trial with a high-intensity constant work-rate endurance test (CWRET) using HFNC and a venturi mask (VM) for oxygen delivery in patients with FILD.

## Methods

### Study population

This prospective randomized controlled crossover trial was performed at Tosei General Hospital in Japan from April 2016. This study was carried out in accord with the principles of the Declaration of Helsinki and approved by the Tosei General Hospital Institutional Review Board (IRB No. 554). The trial was registered in the university hospital medical information network Clinical Trial Registry (UMIN-CTR) (UMIN000021901). All participants provided written informed consent before participation.

Eligible patients were aged ≥18 years who had been diagnosed with FILD in accordance with the previously established criteria [1, 2]. Exclusion criteria were SpO_2_ > 88% during the baseline CWRET, need for high concentration oxygen at rest (FiO_2_ > 50%), coexistence of chronic obstructive pulmonary disease (COPD) (forced expiratory volume in 1 sec [FEV1] / forced vital capacity [FVC] <  0.70), pneumothorax, pneumomediastinum, an unstable disease, and a history of acute exacerbation within the last 1 month.

### Study design

The high-intensity CWRET is considerably more responsive than incremental exercise tests or the 6-min walking test to assess the effects of interventions [14, 15]. According to these studies, we selected the high-intensity CWRET to assess the efficacy of HFNC on exercise capacity.

Based on previous studies, a sample size of 16 patients with randomization was required to detect a mean difference in the endurance time of 2 min between VM and HFNC (8 patients in each group), with a power of 80% at a two-sided alpha level of 0.05 [16, 17]. Considering an expected dropout rate of 10% in a previous exercise training study of FILD patients, a total of 20 patients were recruited [16].

### Randomization and interventions

**Fig. 1:**
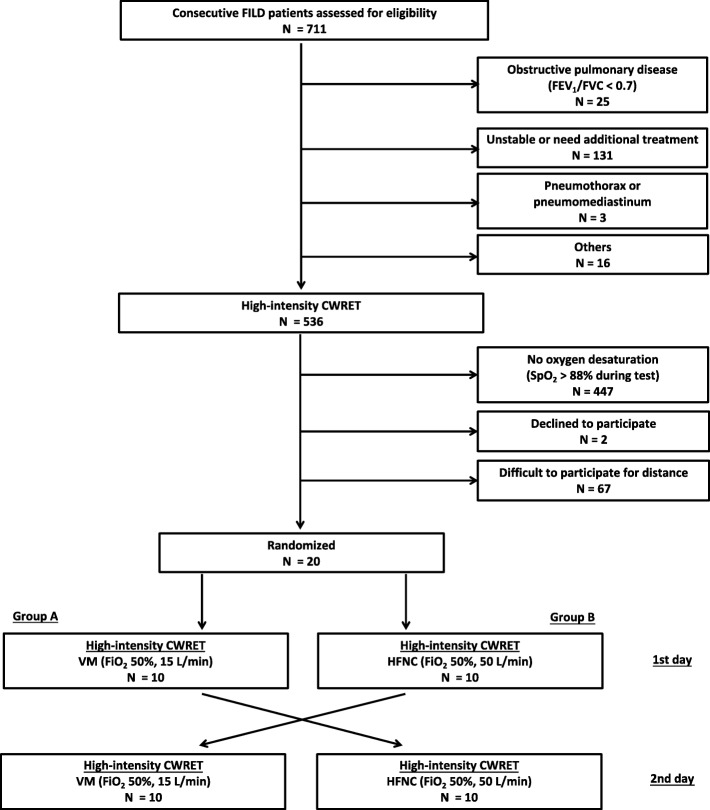
Patient flow chart

Eligible patients first performed a symptom limited incremental exercise test to evaluate the patient’s maximal exercise capacity, using electrically braked cycloergometer (AEROBIKE 800 / AEROBIKE 75XL III; Combi corporation, Tokyo, Japan). The incremental test was performed on room air or in each oxygen flow at rest according to the American Thoracic Society (ATS) /American College of Chest Physicians (ACCP) statement [18]. On another day, patients performed a high-intensity CWRET with 80% of the maximum work-load determined by symptom limited incremental exercise test. Subjects continued at a pedaling rate of 60 cycles/min until they could no longer continue. Patients with SpO_2_ > 88% during the CWRET were excluded before randomization (Fig. 1).

After screening, we finally recruited a total of 20 patients. All patients were randomly allocated into two groups using the block randomization technique (blocks of four patients) in a 1:1 ratio. In group A, a high-intensity CWRET using VM was performed on the first day, and a test using HFNC was performed on the following day. In group B, a high-intensity CWRET using HFNC was performed on the first day, and a test using VM was performed on the following day. The setting for VM (Silente O_2_ Venturi, Intersurgical Ltd., Berkshire, UK) was a FiO_2_ of 50%, with an oxygen flow of 15 L/min. The setting for HFNC (PMB-5000 and PMH 7000 PLUS, Pacific Medico Co., Ltd., Tokyo, Japan) was a FiO_2_ of 50%, with a humidified oxygen flow of 50 l/min. Endurance time was measured on each test. SpO_2_ and heart rate (HR) were monitored throughout the test by pulse oximetry (PULSOX-3, Konica Minolta Japan, Inc., Tokyo, Japan). Subjects were asked to rate their dyspnea and leg fatigue every minute during the test using the modified Borg scale. Isotime values for dyspnea and leg fatigue were defined at the point of termination of the shorter CWRET [15]. Patient’s comfort with each device was assessed at the end of each trial, using a 0 to 10 numerical rating scale (10 = no discomfort).

### Study outcomes

The primary outcome variable was endurance time. The secondary outcome variables were SpO_2_, HR, modified Borg scale, patient’s comfort with each device, and adverse events during endurance exercise test.

### Statistical analysis

Categorical variables were summarized by frequency. Continuous variables were expressed as mean ± standard deviation (SD) or mean (95% confidence interval [CI]). For comparisons with the data for categorical variables or continuous variables between groups, a chi-squared test or Student’s t-test were used. A generalized linear mixed-effects model was applied for both primary and secondary endpoints; the model included device, sequence, and period as fixed effects, and subject within sequence as a random effect. The Bonferroni post-hoc test was performed for multiple comparisons of groups. As a sub-analysis, we compared VM and HFNC data with baseline CWRET. A CWRET good responder was defined as a patient with > 100 s or 33% improvement of endurance time from baseline CWRET [14]. Subgroup analyses of endpoints in VM/HFNC good responders were conducted. We also investigated the relationship between VM/HFNC non-responders and pulmonary hypertension (PH) assessed by either echocardiography (right ventricular systolic pressure > 35 mmHg) or right heart catheterization (mean pulmonary artery pressure ≥ 25 mmHg) [19, 20]. All tests were performed at a significance level of *p* <  0.05. Analysis was completed using IBM SPSS statistics ver. 21 (IBM Corp. Armonk, NY, USA).

## Results

### Baseline characteristics

Between April 2016 and June 2017, 711 patients with FILD were screened for eligibility. After screening, 20 patients were enrolled and randomized for the prospective crossover trial as planned (Fig. 1). The characteristics of the 20 patients are summarized in Table 1. There were no significant differences in any of the data between group A and group B. There were also no significant differences in any of the data between IPF and non-IPF-FILD cases (Supplementary Table S1). All patients completed the trial.

**Table 1:** Patient characteristics

	All	Group A	Group B	*P* value
Number	20	10	10	
Age, years	70.7 ± 7.6	73.0 ± 7.6	68.5 ± 7.2	0.192
Sex, M/F	19/1	10/0	9/1	0.305
BMI, Kg/m^2^	22.3 ± 5.1	20.7 ± 2.7	24.0 ± 6.4	0.149
Smoking status				
Ever/Never	19/1	9/1	10/0	0.305
Pack-years	53.7 ± 40.4	41.8 ± 30.7	65.6 ± 46.8	0.196
mMRC	2.7 ± 1.0	2.8 ± 0.9	2.6 ± 1.2	0.676
FVC, %pred.	60.0 ± 14.7	62.9 ± 19.2	57.0 ± 8.4	0.386
FEV_1_/FVC, %	88.0 ± 8.6	88.5 ± 7.7	87.6 ± 9.8	0.821
DLco, %pred.*	32.5 ± 15.2	32.3 ± 15.0	32.8 ± 16.4	0.953
RV, %pred.**	59.7 ± 23.9	66.8 ± 27.2	52.8 ± 19.4	0.258
Oxygen therapy, Yes	8 (40%)	3 (30%)	5 (50%)	0.361
Flow at rest, L/min	2.2 ± 1.5	2.5 ± 2.3	2.0 ± 1.0	
PaCO_2_, torr	43.2 ± 5.4	44.2 ± 5.6	42.3 ± 5.2	0.433
FILD classification, n				
IPF	12	5	7	
NSIP	1	1	0	
CTD-ILD	2	1	1	
Unclassifiable IIP	5	3	2	
Pulmonary hypertension***	12 (60%)	6 (60%)	6 (60%)	1.000
RVSP ≥35 mmHg	10/15	5/6	5/9	
MPAP ≥25 mmHg	6/18	4/9	2/9	
Baseline CWRET				
Endurance time, min	3.9 ± 3.3	3.5 ± 1.1	4.4 ± 4.6	0.518
Min SpO_2_, %	77.7 ± 6.6	76.9 ± 7.4	78.5 ± 5.9	0.601
Max HR, bpm	121.2 ± 17.9	128.0 ± 11.4	114.3 ± 21.0	0.086
Final Borg scale				
Dyspnea	6.9 ± 2.1	7.2 ± 1.9	6.5 ± 2.3	0.468
Leg fatigue	6.2 ± 2.8	7.0 ± 1.9	5.4 ± 3.3	0.188

Group A: venturi mask (VM) → high-flow nasal cannula (HFNC)

Group B: HFNC → VM

Data are presented as number (%) or mean ± standard deviation (SD)

*P*-value from chi-squared test or Student’s t-test

* *n* = 17 (A: *n* = 9, B: *n* = 8), ** *n* = 16 (A: *n* = 8, B: *n* = 8), *** Pulmonary hypertension was assessed by echocardiography (right ventricular systolic pressure > 35 mmHg) or right heart catheterization (mean pulmonary artery pressure ≥ 25 mmHg)

*BMI* body mass index, *CTD* connective tissue disease, *CWRET* constant work-rate endurance test, *DLco* diffusion capacity for carbon monoxide, *FEV*_*1*_ forced expiratory volume in 1 s, *FVC* forced vital capacity, *IIP* idiopathic interstitial pneumonia, *FILD* fibrotic interstitial lung disease, *HR* heart rate, *IPF* idiopathic pulmonary fibrosis, *mMRC* the modified Medical Research Council dyspnea scale, *MPAP* mean pulmonary artery pressure, *NSIP* non-specific interstitial pneumonia, *PaO*_*2*_ partial pressure of oxygen, *PaCO*_*2*_ partial pressure of carbon dioxide, *RV* residual volume, *RVSP* right ventricular systolic pressure

### Primary and secondary outcomes

**Fig. 2:**
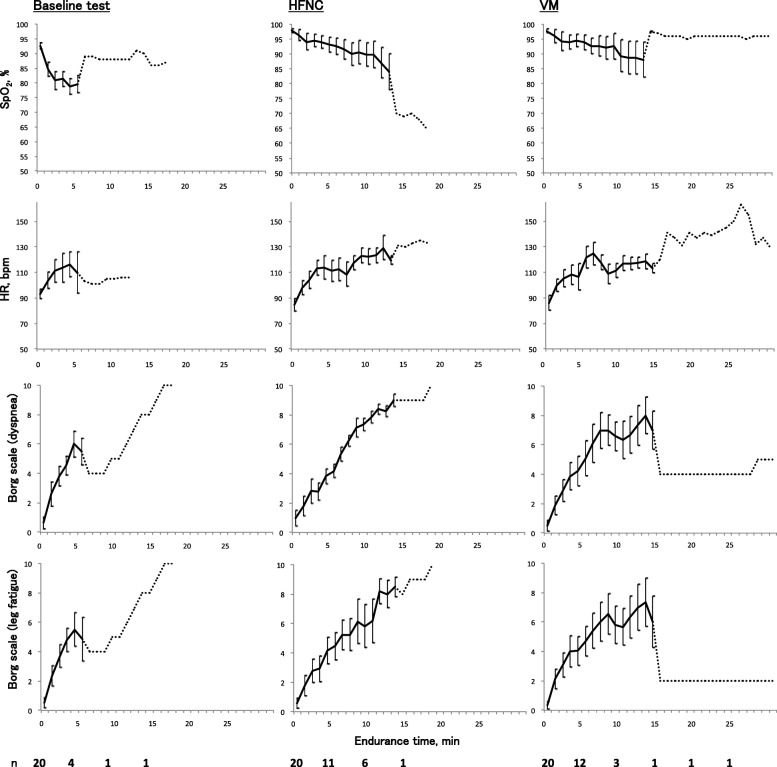
Trend graph of each variable during CWRET. Horizontal axis = endurance time; Vertical axis = individual variable (SpO_2_, heart rate [HR], Borg dyspnea scale, and Borg leg fatigue scale); Solid line = mean value (error bar represents 95% CI); Dotted line = only 1 patient

The median duration between the baseline CWRET and randomization was 2 days (interquartile range 2–7 days). Table 2 shows the comparison of primary and secondary endpoints between VM and HFNC. Compared with the baseline CWRET, the majority of patients were good responders to VM and HFNC (VM *n* = 15, 75%; HFNC *n* = 13, 65%) (Table 2). HFNC did not exceed the efficacy of VM in endurance time (HFNC 6.8 [95% confidence interval (CI) 4.3–9.3] min vs VM 7.6 [95% CI 5.0–10.1] min, *p* = 0.669). There were no significant differences in other secondary endpoints. Trend graph of each variable during endurance exercise tolerance test are shown in Fig. 2 (the data of each subject are shown in supplementary Figure S1, S2, S3 and S4). Two patients complained about nasal pain from HFNC, but it improved immediately. No other adverse events were observed.

**Table 2:** Primary and secondary endpoints (VM vs HFNC)

	VM	HFNC	Difference (95% CI)	*P*-value^**^
Good responder^*^	15 (75%)	13 (65%)		
Primary endpoint				
Endurance time, min	7.6 (5.0–10.1)	6.8 (4.3–9.3)	− 0.8 (− 4.4–2.8)	0.669
Secondary endpoint				
Min SpO_2_, %	89.4 (85.1–93.7)	89.7 (85.3–94.0)	0.3 (− 5.8–6.3)	0.934
Max HR, bpm	124.2 (115.9–132.4)	120.8 (112.5–129.1)	−3.8 (− 11.7–4.2)	0.345
Isotime Borg scale (dyspnea)	5.9 (4.6–7.1)	5.9 (4.7–7.1)	0.1 (− 1.7–1.8)	0.955
Isotime Borg scale (leg fatigue)	5.7 (4.2–7.2)	5.4 (3.9–6.8)	− 0.3 (− 2.4–1.8)	0.757
Final Borg scale (dyspnea)	7.0 (5.8–8.1)	6.6 (5.4–7.8)	− 0.4 (− 2.0–1.3)	0.672
Final Borg scale (leg fatigue)	6.4 (4.9–7.9)	6.3 (4.8–7.8)	− 0.1 (− 2.3–2.1)	0.926
Patient comfort of device	7.8 (6.7–8.9)	6.3 (5.2–7.4)	− 1.5 (− 3.1–0.1)	0.067

Data are presented as number (%) or mean (95% CI)

*HFNC* high-flow nasal cannula, *HR* heart rate, *SpO*_*2*_ saturation of peripheral oxygen, *VM* venturi mask

^*^Good responder was defined as a patient with > 100 s or 33% improvement of endurance time from baseline CWRET,

^**^Calculated by generalized linear mixed-effects model with fixed factors for each device, sequence, and period, and a random factor for subject within sequence

### Sub-analysis compared with the baseline CWRET

As a sub-analysis, we compared VM and HFNC data with baseline CWRET (Table 3). Compared with the baseline test, both VM and HFNC significantly improved min SpO_2_ (baseline 77.7%; VM 89.4%; HFNC 89.7%) and isotime Borg dyspnea scale (baseline 6.6; VM 4.5; HFNC 3.9). HFNC also improved isotime Borg leg fatigue scale (baseline 6.0, HFNC 3.7).

**Table 3:** Sub-analysis compared with the baseline CWRET

	Mean	95% CI	*P*-value^*^
Endurance time, min			
Baseline test	3.9	1.7–6.2	Ref.
VM	7.6	5.3–9.8	0.076
HFNC	6.8	4.6–9.0	0.226
Min SpO_2_, %			
Baseline test	77.7	73.8–81.6	Ref.
VM	89.4	85.5–93.3	< 0.001
HFNC	89.7	85.8–93.5	< 0.001
Max HR, bpm			
Baseline test	121.2	113.2–129.1	Ref.
VM	124.2	116.2–132.1	1.000
HFNC	120.8	112.8–128.8	1.000
Isotime Borg scale (dyspnea)			
Baseline test	6.6	5.4–7.7	Ref.
VM	4.5	3.4–5.6	0.040
HFNC	3.9	2.8–5.1	0.006
Isotime Borg scale (leg fatigue)			
Baseline test	6.0	4.7–7.3	Ref.
VM	4.3	3.0–5.6	0.188
HFNC	3.7	2.4–4.9	0.035
Final Borg scale (dyspnea)			
Baseline test	6.9	5.8–7.9	Ref.
VM	7.0	5.9–8.0	1.000
HFNC	6.6	5.5–7.7	1.000
Final Borg scale (leg fatigue)			
Baseline test	6.2	4.7–7.6	Ref.
VM	6.4	5.0–7.8	1.000
HFNC	6.3	4.9–7.7	1.000

^*^Calculated by generalized linear mixed-effects model with fixed factors for each device, sequence, and period, and a random factor for subject within sequence. The Bonferroni post-hoc test was performed for multiple comparisons of groups

### Subgroup analysis in HFNC good responders

Among HFNC good responders (*n* = 13), the majority were also VM good responders (*n* = 11, 85%). In this subgroup, HFNC was superior in 6 patients, VM was superior in 1 patient, and the two were equivalent in 6 patients (superior: > 100 s or 33% improvement in endurance time). HFNC clearly reduced exertional dyspnea compared to VM in some patients (Case 1, 11, 12, 16, 19) (Supplementary Figure S3). Subgroup analysis of HFNC responders revealed that HFNC significantly extended the endurance time compared with VM (VM 6.4 [95%CI 4.5–8.3] min vs HFNC 7.8 [95%CI 5.8–9.7] min, *p* = 0.046), while no similar effect was observed in the analysis of VM responders (Table 4, Supplementary Table S2). No significant differences were found in baseline characteristics between HFNC good responders and non-responders (Supplementary Table S3).

**Table 4:** Subgroup analysis of endpoints in HFNC good responders

*N* = 13	VM	HFNC	Difference (95% CI)	*P*-value^**^
Primary endpoint				
Endurance time, min	6.4 (4.5–8.3)	7.8 (5.8–9.7)	1.4 (0.0–2.7)	0.046
Secondary endpoint				
Min SpO_2_, %	89.7 (84.3–95.0)	88.6 (83.2–93.9)	−1.1 (−3.4–1.2)	0.319
Max HR, bpm	124.2 (112.4–136.1)	120.5 (108.6–132.4)	−3.7 (− 14.8–7.4)	0.478
Isotime Borg scale (dyspnea)	6.7 (5.3–8.1)	6.4 (5.0–7.8)	−0.3 (− 1.6–1.0)	0.632
Isotime Borg scale (leg fatigue)	6.3 (4.5–8.1)	6.0 (4.2–7.7)	− 0.4 (− 1.8–1.1)	0.583
Final Borg scale (dyspnea)	7.3 (5.8–8.8)	7.2 (5.7–8.6)	− 0.2 (− 0.5–0.2)	0.153
Final Borg scale (leg fatigue)	6.6 (4.7–8.6)	6.9 (4.9–8.8)	0.3 (− 0.8–1.4)	0.614
Patient comfort of device	8.4 (7.2–9.6)	7.3 (6.1–8.5)	− 1.0 (−2.7–0.7)	0.220

Data are presented as number (%) or mean (95% CI)

*HFNC* high-flow nasal cannula, *HR* heart rate, *SpO*_*2*_ saturation of peripheral oxygen, *VM* venturi mask

^*^Good responder was defined as a patient with > 100 s or 33% improvement in endurance time from baseline CWRET,

^**^Calculated by generalized linear mixed-effects model with fixed factors for each device, sequence, and period, and a random factor for subject within sequence

### Relationship between VM/HFNC responders and PH

Echocardiography or right heart catheterization was performed in all patients, and 12 patients were diagnosed with PH (Table 1). There was no significant difference in the proportion of HFNC non-responders between patients with and without PH (PH 33% vs non-PH 38%; chi-squared test, *p*-value 0.848). Additionally, there was no significant difference in the proportion of VM non-responders between patients with and without PH (PH 33% vs non-PH 12%; chi-squared test, *p*-value 0.292).

## Discussion

To the best of our knowledge, this is the first randomized crossover trial of an exercise test using HFNC in FILD patients. Contrary to our expectations, this study did not meet the prespecified endpoints. However, the majority of patients responded well to HFNC, and the effect was superior to VM in some patients. Moreover, HFNC significantly extended the endurance time compared with VM in the subgroup analysis. Although we could not prove its efficacy due to the small sample size and unchanged settings, HFNC oxygen therapy may improve exercise capacity in FILD patients.

HFNC clearly improved endurance time and exertional dyspnea in a limited number of patients (Case 1, 11, 12, 16, 19). We may conjecture several potential mechanisms for these beneficial effects. First, washout of the physiological dead space may have improved patients’ work of breathing. Bräunlich et al. [21] reported that HFNC decreases respiratory rate and carbon dioxide (CO_2_) levels in patients with IPF and COPD. A recent randomized controlled crossover trial also showed that HFNC decreases respiratory rate and CO_2_ levels in stable COPD patients [22]. These beneficial effects may have contributed to improving exercise capacity and exertional dyspnea. Second, improvement in mucosal dryness with heated and humidified oxygen may have improved patient comfort during exercise. Chanques et al. [23] reported that under-humidified high-flow oxygen therapy was associated with patients’ discomfort and mouth-throat dryness. HFNC can deliver heated and humidified oxygen, which may have led to greater comfort during exercise. Finally, positive airway pressure may have improved dynamic hyperinflation and alveolar collapse. A previous report showed that HFNC increases airway pressure as flow increases [24]. In a recent clinical study, HFNC improved the tidal volume and end-expiratory lung volume compared with conventional oxygen therapy in COPD patients [13]. Even though we excluded patients with concurrent airflow limitations (FEV1/FVC <  0.7), these effects might be associated with the improvement of oxygenation and exercise capacity. Unfortunately, we could not assess physiological variables including end-expiratory pressure and end tidal CO_2_. Future researches may reveal the exact mechanisms.

Conversely, we found that HFNC was inferior to VM in a few patients (Case 6, 7, 9). A recent experimental study using an airway model made with a 3D printer demonstrated that increasing the flow rate of HFNC generates higher positive end-expiratory pressure (PEEP) but does not necessarily increase the washout effects [25]. In our study, two patients complained about nasal pain, although it improved immediately. We suppose that the flow rate of 50 L/min might have been too strong for them. Although the flow rate was determined with reference to previous studies, further validation studies will be required [10–12].

Differences in pathophysiology between FILD and COPD should be considered. Although the pathophysiology of both diseases is complex, FILD is mainly affected by restrictive impairment, while COPD is mainly affected by airflow limitation. Previous studies have demonstrated that non-invasive ventilatory support by continuous positive airway pressure (CPAP) and pressure support ventilation (PSV) improves exercise performance and exertional dyspnea in COPD patients [26, 27]. On the other hand, Moderno et al. [28] showed that CPAP did not improve exercise performance compared with proportional assist ventilation (PAV) in IPF patients. Considering these findings, a decrease in the work of breathing may be more important than an increase in PEEP in improving the exercise capacity in FILD patients. The most appropriate individual settings to decrease the work of breathing may improve the exercise performance of HFNC non-responders.

In the present study, both VM and HFNC significantly improved oxygenation and exertion dyspnea compared with the baseline test. There was no significant difference in the proportion of responders between patients with and without PH. Our findings emphasize the importance of supplemental oxygen for improving exercise capacity in FILD patients. Furthermore, HFNC has been reported to improve health status and exercise capacity in COPD patients within a mid-long-term [29, 30]. An exercise training significantly improved exercise capacity and health status in patients with FILD [31–33]. Combining appropriate oxygen therapy with exercise training may enhance its effectiveness in FILD patients.

Our study has some limitations. First, this is a small single-center study. Second, the heterogeneity of FILD needs to be considered. Recent clinical studies have demonstrated the similarities in genetics, pathophysiology, and clinical course between IPF and non-IPF-FILD [34–36]. Additionally, previous reports showed a reduced exercise capacity in patients with non-IPF-FILD, similar to that in patients with IPF [37, 38]. Considering these findings, we recruited patients with all forms of FILD. Third, the carryover effects may need to be considered. To minimize the potential bias and carryover effects, we used a generalized linear mixed-effects model adjusted for device, sequence, and period as fixed effects, and subject within sequence as a random effect. Finally, gender split and racial differences may need to be considered since most patients were male and all were Japanese.

## Conclusions

We first examined the efficacy of HFNC oxygen therapy on exercise capacity in FILD patients. In this study, HFNC did not exceed the efficacy of VM, however, it may be beneficial if the settings match. Predetermining comfortable settings for each patient may increase the effectiveness of HFNC. Further studies will be required to confirm the potential benefits of HFNC for improving exercise capacity in FILD patients.

## Supplementary information

**Additional file 1: Table S1**. Comparison of baseline characteristics between IPF and non-IPF. **Table S2**. Subgroup analysis of endpoints in VM good responders. **Table S3.** Comparison of baseline characteristics between HFNC good responders and non-responders. **Figure S1.** Trend graph of SpO_2_ during endurance exercise tolerance test in each subject. **Figure S2**. Trend graph of heart rate (HR) during endurance exercise tolerance test in each subject. **Figure S3**. Trend graph of Borg scale (dyspnea) during endurance exercise tolerance test in each subject. **Figure S4**. Trend graph of Borg scale (leg fatigue) during endurance exercise tolerance test in each subject.

## Data Availability

All data generated or analyzed during this study are included in this published article and its supplementary information files.

## Abbreviations

COPD: Chronic obstructive pulmonary disease
CPAP: Continuous positive airway pressure
CWRET: Constant work-rate endurance test
FEV_1_: Forced expiratory volume in one second
FILD: Fibrotic interstitial lung disease
FVC: Forced vital capacity
HFNC: High-flow nasal cannula
IIP: Idiopathic interstitial pneumonia
IPF: Idiopathic pulmonary fibrosis
NSIP: Non-specific interstitial pneumonia
PEEP: Positive end-expiratory pressure
PH: Pulmonary hypertension
VM: Venturi mask
