# Supplementary material for: The impact of high-flow nasal cannula oxygen therapy on exercise capacity in fibrotic interstitial lung disease: a proof-of-concept randomized controlled crossover trial
Source: BMC Pulm Med. 2020 Feb 24;20:51. doi: 10.1186/s12890-020-1093-2 (PMC7041255; doi:10.1186/s12890-020-1093-2)

**SUPPLEMENTARY INFORMATION**

**Title**

The impact of high-flow nasal cannula oxygen therapy on exercise capacity in fibrotic interstitial lung disease: a proof-of-concept randomized controlled crossover trial

**Authors**

Atsushi Suzuki^1,2^, Masahiko Ando^3^, Tomoki Kimura^1^, Kensuke Kataoka^1^, Toshiki Yokoyama^1^, Eiichi Shiroshita^4^ and Yasuhiro Kondoh^1^

**Affiliations**

^1^Department of Respiratory Medicine and Allergy, Tosei General Hospital, Seto, Aichi, Japan

^2^Department of Respiratory Medicine, Nagoya University Graduate School of Medicine, Nagoya, Aichi, Japan

^3^Center for Advanced Medicine and Clinical Research, Nagoya University

Hospital, Nagoya, Aichi, Japan

^4^Pacific Medico Co., Ltd., Chiyoda-ku, Tokyo, Japan

**Table S1. Comparison of baseline characteristics between IPF and non-IPF**

|  | IPF | Non-IPF-FILD | P value |
| --- | --- | --- | --- |
| Number | 12 | 8 |  |
| Age, years | 69.4 ± 5.4 | 72.8 ± 10.1 | 0.349 |
| Sex, M/F | 11/1 | 8/0 | 0.402 |
| BMI, Kg/m^2^ | 21.6 ± 2.8 | 23.4 ± 7.4 | 0.449 |
| Smoking status |  |  |  |
| Ever/Never | 11/1 | 8/0 | 0.402 |
| Pack-years | 51.9 ± 47.9 | 56.3 ± 28.7 | 0.822 |
| mMRC | 2.8 ± 1.1 | 2.5 ± 0.9 | 0.494 |
| FVC, %pred. | 56.4 ± 13.4 | 65.2 ± 15.9 | 0.198 |
| FEV_1_/FVC, % | 88.0 ± 7.9 | 88.1 ± 7.9 | 0.981 |
| DLco, %pred.* | 28.0 ± 11.6 | 37.5 ± 17.9 | 0.209 |
| RV, %pred.** | 51.5 ± 23.1 | 68.9 ± 23.1 | 0.173 |
| Oxygen therapy, Yes |  |  |  |
| Flow at rest, L/min | 0.9 ± 1.2 | 0.8 ± 1.7 | 0.877 |
| PaCO_2_, torr | 44.3 ± 4.9 | 41.6 ± 6.0 | 0.286 |
| Pulmonary hypertension | 6 | 6 | 0.264 |
| Baseline CWRET |  |  |  |
| Endurance time, min | 3.1 ± 1.3 | 5.3 ± 4.9 | 0.145 |
| Min SpO_2_, % | 77.2 ± 5.6 | 78.5 ± 8.2 | 0.669 |
| Max HR, bpm | 120.2 ± 18.5 | 122.6 ± 18.0 | 0.772 |
| Final Borg scale |  |  |  |
| Dyspnea | 6.8 ± 2.3 | 7.0 ± 1.9 | 0.801 |
| Leg fatigue | 5.3 ± 2.9 | 7.5 ± 2.0 | 0.079 |

Data are presented as number (%) or mean ± standard deviation (SD).

P-value from chi-squared test or Student’s t-test.

* n = 17 (A: n = 9, B: n = 8), ** n = 16 (A: n = 8, B: n = 8)

**Table S2. Subgroup analysis of endpoints in VM good responders**

| N = 15 | VM | HFNC | Difference (95% CI) | P-value^**^ |
| --- | --- | --- | --- | --- |
| **Primary endpoint** |  |  |  |  |
| Endurance time, min | 8.1 (5.0-11.1) | 6.6 (3.6-9.7) | -1.4 (-5.8-2.9) | 0.499 |
| **Secondary endpoint** |  |  |  |  |
| Min SpO_2_, % | 90.2 (85.6-94.8) | 90.5 (85.9-95.3) | 0.3 (-6.3-6.9) | 0.918 |
| Max HR, bpm | 120.0 (110.8-129.2) | 118.3 (109.0-127.3) | -1.8 (-14.8-11.1) | 0.775 |
| Isotime Borg scale (dyspnea) | 5.6 (4.1-7.0) | 5.9 (4.4-7.3) | 0.3 (-1.8-2.4) | 0.774 |
| Isotime Borg scale (leg fatigue) | 4.9 (3.1-6.6) | 4.9 (3.1-6.6) | 0.0 (-2.4-2.5) | 0.997 |
| Final Borg scale (dyspnea) | 6.9 (5.5-8.2) | 6.5 (5.2-7.9) | -0.3 (-2.2-1.6) | 0.710 |
| Final Borg scale (leg fatigue) | 5.6 (3.8-7.3) | 5.5 (3.8-7.3) | -0.0 (-2.5-2.5) | 0.988 |
| Patient comfort of device | 8.0 (6.5-9.4) | 6.3 (4.9-7.8) | -1.6 (-3.6-0.4) | 0.109 |

Data are presented as number (%) or mean (95% CI).

HFNC, high-flow nasal cannula; HR, heart rate; SpO_2_, saturation of peripheral oxygen; VM, venturi mask.

^*^ Good responder was defined as a patient with > 100 seconds or 33 % improvement in endurance time from baseline CWRET.

^**^Calculated by generalized linear mixed-effects model with fixed factors for each device, sequence, and period,

and a random factor for subject within sequence.

**Table S3. Comparison of baseline characteristics between HFNC good responders and non-responders**

|  | HFNC  Responders | HFNC  Non-responders | P value |
| --- | --- | --- | --- |
| Number | 13 | 7 |  |
| Age, years | 71.5 ± 7.7 | 69.3 ± 7.7 | 0.541 |
| Sex, M/F | 13/0 | 6/1 | 0.162 |
| BMI, Kg/m^2^ | 22.0 ± 5.8 | 22.9 ± 3.7 | 0.725 |
| Smoking status |  |  |  |
| Ever/Never | 12/1 | 7/0 | 0.452 |
| Pack-years | 59.8 ± 45.1 | 42.2 ± 29.7 | 0.371 |
| mMRC | 2.8 ± 0.9 | 2.6 ± 1.3 | 0.694 |
| FVC, %pred. | 58.8 ± 16.4 | 62.1 ± 11.7 | 0.650 |
| FEV_1_/FVC, % | 89.5 ± 7.7 | 85.2 ± 10.1 | 0.295 |
| DLco, %pred.* | 34.0 ± 18.5 | 30.4 ± 9.5 | 0.645 |
| RV, %pred.** | 57.9 ± 25.9 | 62.2 ± 22.9 | 0.739 |
| Oxygen therapy, Yes |  |  |  |
| Flow at rest, L/min | 0.7 ± 1.0 | 1.2 ± 2.0 | 0.536 |
| PaCO_2_, torr | 42.7 ± 6.1 | 44.2 ± 3.7 | 0.576 |
| FILD classification, n |  |  |  |
| IPF | 9 | 3 |  |
| NSIP | 0 | 1 |  |
| CTD-ILD | 0 | 1 |  |
| Unclassifiable IIP | 4 | 1 |  |
| Baseline CWRET |  |  |  |
| Endurance time, min | 3.3 ± 1.4 | 5.1 ± 5.3 | 0.247 |
| Min SpO_2_, % | 78.5 ± 5.1 | 76.3 ± 9.1 | 0.573 |
| Max HR, bpm | 118.1 ± 16.1 | 126.9 ± 20.9 | 0.307 |
| Final Borg scale |  |  |  |
| Dyspnea | 6.9 ± 2.3 | 6.9 ± 1.8 | 0.991 |
| Leg fatigue | 6.6 ± 2.3 | 5.4 ± 3.5 | 0.344 |

Data are presented as number (%) or mean ± standard deviation (SD).

P-value from chi-squared test or Student’s t-test.

* n = 17 (A: n = 9, B: n = 8), ** n = 16 (A: n = 8, B: n = 8)

**Figure S1. Trend graph of SpO_2_ during endurance exercise tolerance test in each subject**

**

**Dotted line = baseline test; Dashed line = VM; Solid line = HFNC

**Figure S2. Trend graph of heart rate (HR) during endurance exercise tolerance test in each subject**

**
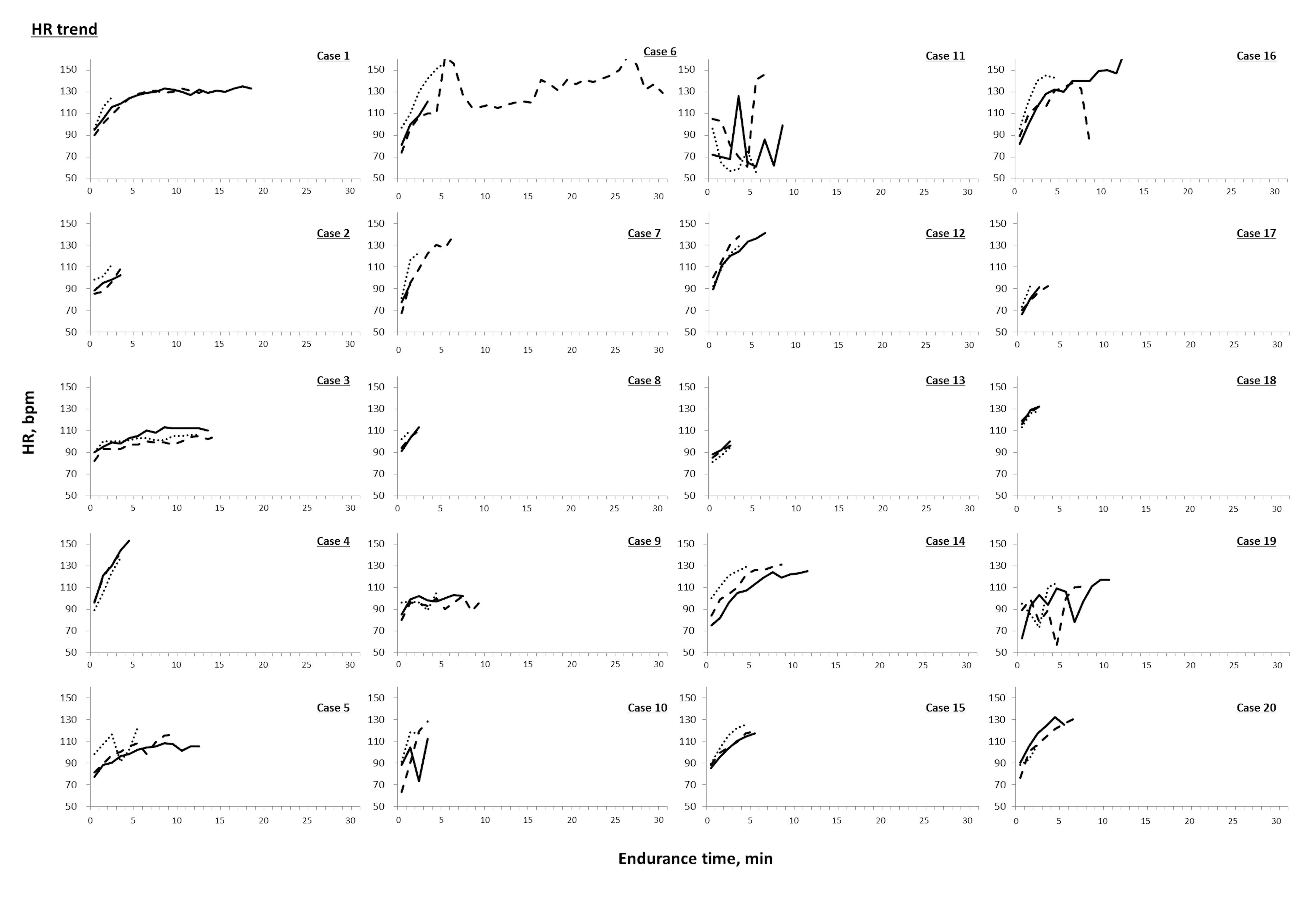
**

**Figure S3. Trend graph of Borg scale (dyspnea) during endurance exercise tolerance test in each subject**

**
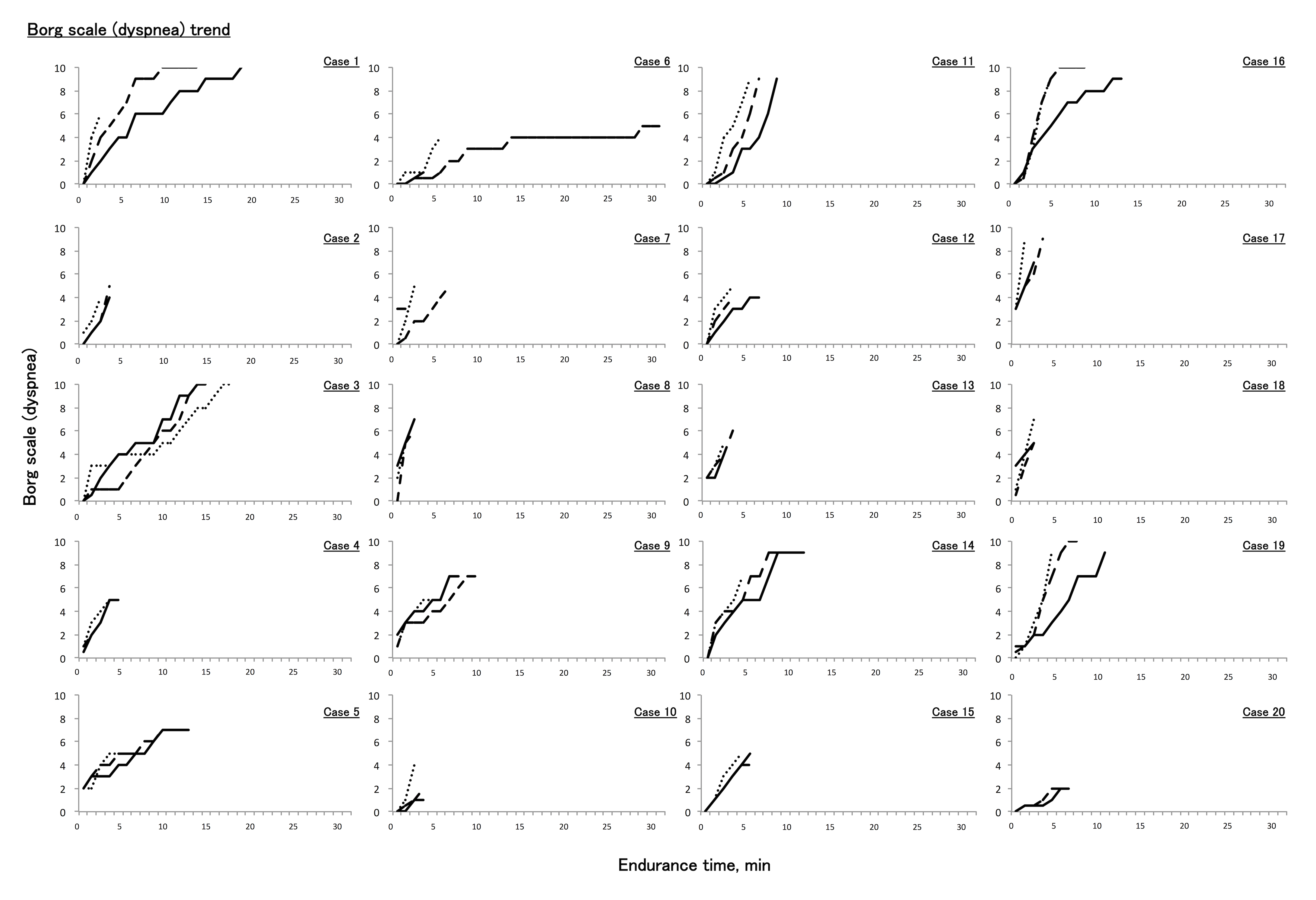
**

**Figure S4. Trend graph of Borg scale (leg fatigue) during endurance exercise tolerance test in each subject**


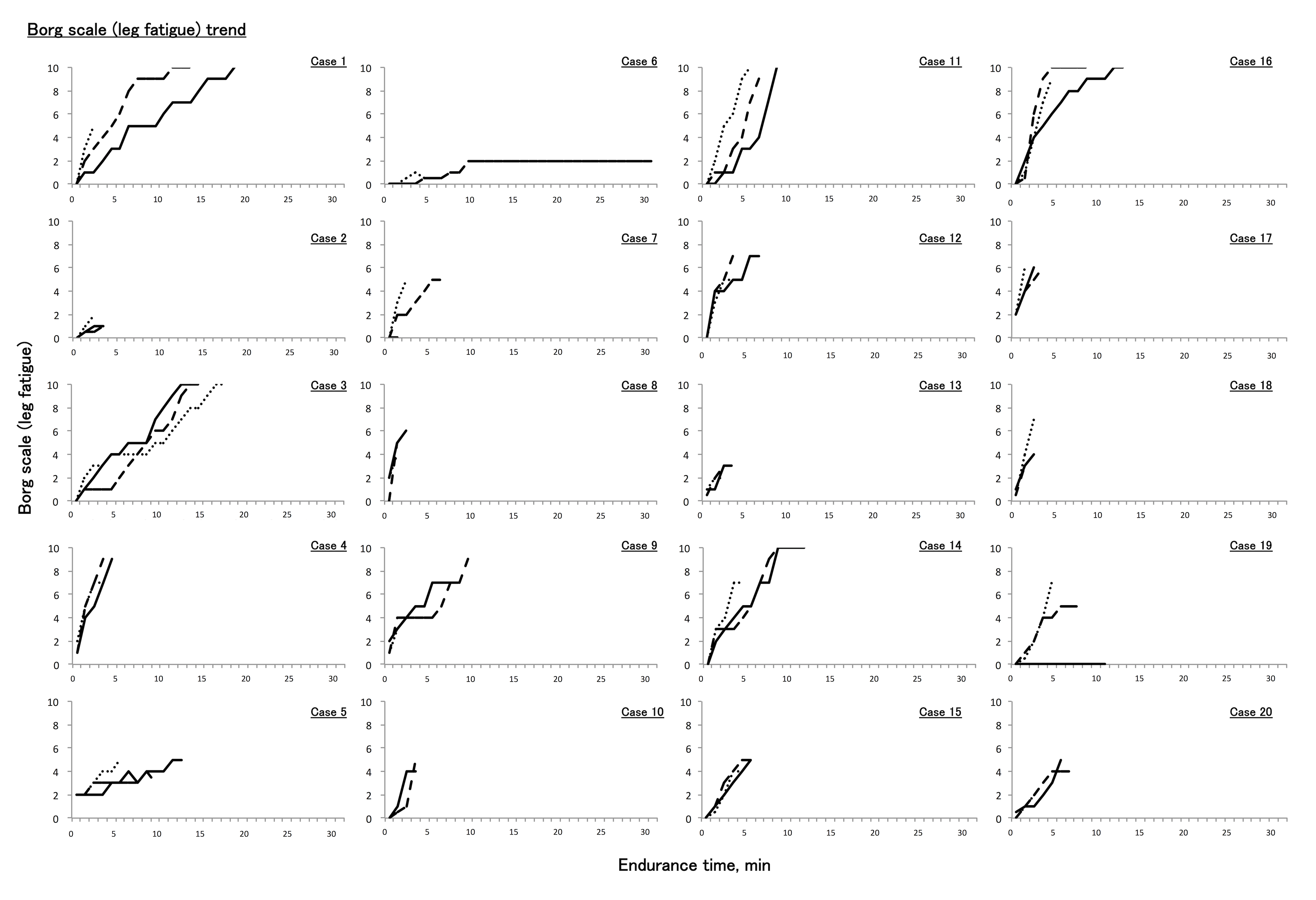

Supplement: Supplementary file 1 — Additional file 1: Table S1. Comparison of baseline characteristics between IPF and non-IPF. Table S2. Subgroup analysis of endpoints in VM good responders. Table S3. Comparison of baseline characteristics between HFNC good responders and non-responders. Figure S1. Trend graph of SpO2 during endurance exercise tolerance test in each subject. Figure S2. Trend graph of heart rate (HR) during endurance exercise tolerance test in each subject. Figure S3. Trend graph of Borg scale (dyspnea) during endurance exercise tolerance test in each subject. Figure S4. Trend graph of Borg scale (leg fatigue) during endurance exercise tolerance test in each subject. [file 12890_2020_1093_MOESM1_ESM.docx]
